# Effect of Frustration on Brain Activation Pattern in Subjects with Different Temperament

**DOI:** 10.3389/fpsyg.2015.01989

**Published:** 2016-01-11

**Authors:** Maria Bierzynska, Maksymilian Bielecki, Artur Marchewka, Weronika Debowska, Anna Duszyk, Wojciech Zajkowski, Marcel Falkiewicz, Anna Nowicka, Jan Strelau, Malgorzata Kossut

**Affiliations:** ^1^Laboratory of Neuroplasticity, Department of Molecular and Cellular Neurobiology, Nencki Institute of Experimental BiologyWarsaw, Poland; ^2^Department of Psychology, SWPS University of Social Sciences and HumanitiesWarsaw, Poland; ^3^Laboratory of Brain Imaging, Neurobiology Center, Nencki Institute of Experimental BiologyWarsaw, Poland; ^4^Laboratory of Psychophysiology, Department of Neurophysiology, Nencki Institute of Experimental BiologyWarsaw, Poland

**Keywords:** frustration, temperament, tactile discrimination, fMRI, individual differences, stress

## Abstract

In spite of the prevalence of frustration in everyday life, very few neuroimaging studies were focused on this emotional state. In the current study we aimed to examine effects of frustration on brain activity while performing a well-learned task in participants with low and high tolerance for arousal. Prior to the functional magnetic resonance imaging session, the subjects underwent 2 weeks of Braille reading training. Frustration induction was obtained by using a novel highly difficult tactile task based on discrimination of Braille-like raised dots patterns and negative feedback. Effectiveness of this procedure has been confirmed in a pilot study using galvanic skin response and questionnaires. Brain activation pattern during tactile discrimination task before and after frustration were compared directly. Results revealed changes in brain activity in structures mostly reported in acute stress studies: striatum, cingulate cortex, insula, middle frontal gyrus and precuneus and in structures engaged in tactile Braille discrimination: SI and SII. Temperament type affected activation pattern. Subjects with low tolerance for arousal showed higher activation in the posterior cingulate gyrus, precuneus, and inferior parietal lobule than high reactivity group. Even though performance in the discrimination trials following frustration was unaltered, we observed increased activity of primary and secondary somatosensory cortex processing the tactile information. We interpret this effect as an indicator of additional involvement required to counteract the effects of frustration.

## Introduction

Frustration occurs when an individual continues an action in the expectation of the gratification or desired goal but does not actually attain it (Dollard et al., 1939; Berkowitz, 1989; Anderson and Bushman, 2002). This behavioristic definition dates back to the origins of the frustration-aggression hypothesis in the 1930s (Dollard et al., 1939). The aftermath of a frustrating occurrence may lead to many emotional and affective responses, such as acute stress, lasting anger, sadness, and rage. Those elements, often mixed together in a variety of proportions, constitute frustration. Because of this complexity, frustration is rarely the direct subject of neuroscientific debate and brain imaging studies. Hence, our knowledge of its neural basis is derived mainly from studies focusing on its subcomponents and sequelae, i.e., acute stress and aggression.

Procedures that have been proven to consistently evoke a stress response either induce an atmosphere of high achievement, involve social evaluation or tasks that the subjects have no control over (Dickerson and Kemeny, 2004). In the social context, if negative evaluation is perceived as exclusion, stress response can also be interpreted as frustration (Abler et al., 2005). This further solidifies the similarities between stress and frustration. Not all stress-inducing tasks, however, are likely to lead to frustration. In the psychological review “Criteria of frustration” Britt and Janus (1940) stated that frustration situation must be characterized by the element of barrier or obstruction (Mowrer, 1938) and the expectation of reward or the attainment of a goal (Dollard et al., 1939). Out of commonly used experimental procedures (Dedovic et al., 2009) there are only two that meet these specific criteria - the serial subtraction task (Wang et al., 2005) and the Montreal Stress Imaging Task (MIST; Dedovic et al., 2005). Both of them have also been noted as the only stress-related procedures to induce a significant cortisol stress response that were used in neuroimaging studies (Dedovic et al., 2005, 2009).

A common experimental procedure of the serial subtraction test involves subtracting a given number from the current, much larger one repeatedly – usually subtracting an odd number like 13 or 7 from a four digit starting number (i.e., 2000, 1993, 1986, and so on). During the counting subjects are prompted for faster responses. The control condition is usually much simpler, such as counting backward from 500.

Montreal Stress Imaging Task protocol adapted to a functional magnetic resonance imaging (fMRI) setting by Dedovic et al. (2005) consists of series of computerized mental arithmetic challenges coupled with a social evaluative stress component. The procedure involves three conditions – rest (no task), control (easy task, no evaluation), and experimental (task exceeding mental capacity and negative social evaluation). The negative feedback is delivered both during (onscreen) and between the trials, when the experimenter reminds the participant that his or her results are below the required minimum.

There are several neuroimaging studies employing these paradigms. A positron emission tomography (PET) study in the serial subtraction paradigm (Wang et al., 2005) found significant cerebral blood flow (CBF) increase in the right ventral prefrontal cortex (RVPFC) and left insula/putamen regions that were correlated with the participants’ subjective stress evaluation.

The most consistent stress-related results from MIST studies indicate decreased activity in parts of the limbic system: hippocampus, medio-orbitofrontal cortex (mOFC) and ACC (Pruessner et al., 2010) and increased dopamine release in ventral striatum and basal ganglia (Pruessner et al., 2004; Soliman et al., 2008; Dedovic et al., 2009).

To the best of our knowledge there have been so far only two fMRI studies explicitly dealing with frustration. In the first experiment the authors defined frustration as an emotional component of processing the omission of a desired goal (Abler et al., 2005). The experiment consisted of the series of easy tasks. After each series the participants could either or get nothing. The results revealed a decrease in ventral striatum activity and an increase in RVPFC and right insula in the reward omission condition. In the second study (Yu et al., 2014) authors developed a procedure in which expected reward was blocked and levels of experienced frustration were parametrically varied by manipulating the participants’ motivation to obtain the reward prior to blocking. The activations associated with frustration were observed in amygdala, midbrain periaqueductal gray (PAG), insula, and prefrontal cortex. However, the authors of this study were interested mostly in frustration that leads to reactive aggression.

In our study we introduced a novel procedure evoking frustration that differs in many important aspects from the existing ones. Firstly, our task was based on discrimination of Braille-like characters, hence, it allowed us to study the influence of frustration on activation patterns which are well-defined and thoroughly studied (Sadato et al., 2002). Secondly, our participants had never had any contact with Braille alphabet prior to the experiment and were all provided with the 2 weeks of intensive training in Braille reading. That allowed us to study the effects of frustration in a group of well-trained subjects having equal level of experience and skill in the studied task. Frustration induction was obtained by using a novel tactile task based on discrimination of Braille-like raised dots patterns and negative feedback., Behavioral pre-tests verified that our paradigm was effective in eliciting frustration including both its subjective and physiological components.

The main goal of current fMRI experiment was to study the neural effects of frustration elicited using this paradigm. We hypothesized that structures connected to stress and negative feedback (Dedovic et al., 2009; striatum, insula, and prefrontal regions) will display increased activation pattern after the frustration induction. Furthermore, we suspected that structures associated with tactile discrimination (SI) will be activated bilaterally due to increased attention on given task after the stressor.

To further corroborate the results concerning the influence of frustration, we decided to take into account temperamental differences between participants as potential moderators of brain activation patterns. According to the Regulative Theory of Temperament (RTT; Strelau, 1996, 2008), temperament is defined as basic, primarily biologically determined and relatively stable personality traits, which apply to the formal, energetic and temporal characteristics of reactions and behavior. RTT distinguishes six temperament traits: briskness (BR), perseveration (PE), sensory sensitivity (SS), emotional reactivity (ER), endurance (EN), and activity (AC). RTT postulates that the role of temperament in human adaptation to the environmental conditions is especially evident in stressful situations (Strelau, 2008). When comparing individual differences, we focused primarily on the tolerance for arousal, which is a global temperamental characteristic. This can be done by examining the joined influence of three traits: ER, EN, and AC (Zawadzki and Strelau, 1997). People with low ER, high EN and high AC have a high level of tolerance for stimulation and generally perform well in highly stimulating environments, while the converse is true for persons with the opposite characteristics. This trait is predictive of the psychological well-being (Strelau, 2008) and may impact the response to frustration conditions. In line with the existing studies on moderating role of temperament in stress-response (Strelau, 1996) we expected more pronounced reactions to frustrating manipulation in subjects with low tolerance for arousal. In particular, we expected stronger activations in the regions engaged in processing of emotional reaction.

## Materials and Methods

### Subjects

Twenty nine right-handed sighted subjects (18 women, 11 men, mean age: 23.2 ± 2.4) with no history of neurological or psychiatric disorders, participated in the study. All participants gave their informed consent prior to the start of the experiment. A local research ethics committee at the SWPS University of Social Sciences and Humanities approved the experimental protocol of the study.

### Formal Characteristics of Behavior – Temperament Inventory (FCB-TI)

Formal Characteristics of Behavior – Temperament Inventory (FCB-TI; Zawadzki and Strelau, 1997) is a self-report questionnaire. It consists of 120 items in the form of statements to which the subject responds YES or NO. The items are grouped into six scales: Briskness (Cronbach’s α = 0.77), Perseveration (Cronbach’s α = 0.79), Sensory Sensitivity (Cronbach’s α = 0.73), Emotional Reactivity (Cronbach’s α = 0.83), Endurance (Cronbach’s α = 0.85), and Activity (Cronbach’s α = 0.83). The RTT elaborated by Strelau provides the theoretical framework for the FCB-TI. Study participants were selected from the larger pool consisting of 80 subjects that filled out FCB-TI questionnaire in the preliminary recruitment. Tolerance for stimulation was computed for each subject as an average combining Activity, Endurance, and reversed Emotional Reactivity scores (Zawadzki and Strelau, 1997). Based on these results we selected two groups characterized by extreme scores: 15 participants with lowest and 14 participants with highest tolerance for arousal.

### Braille Reading Training

Braille reading training consisted of 10 supervised Braille reading lessons (30 min per day) conducted over the course of 2 weeks. Subjects were trained to use only their index finger of the right hand while reading. They kept their eyes open during the trainings and they were asked to look straight ahead and not at the Braille text. They learned to read consecutive characters of Braille, separately, in words and in sentences, only by touch. Special Braille handbook was used for the purpose of the training (Andraszewski, 2006). Procedure of training was consulted and supervised by professional Braille teachers from Polish Association for the Blind^1^

### fMRI Task

**FIGURE 1:**
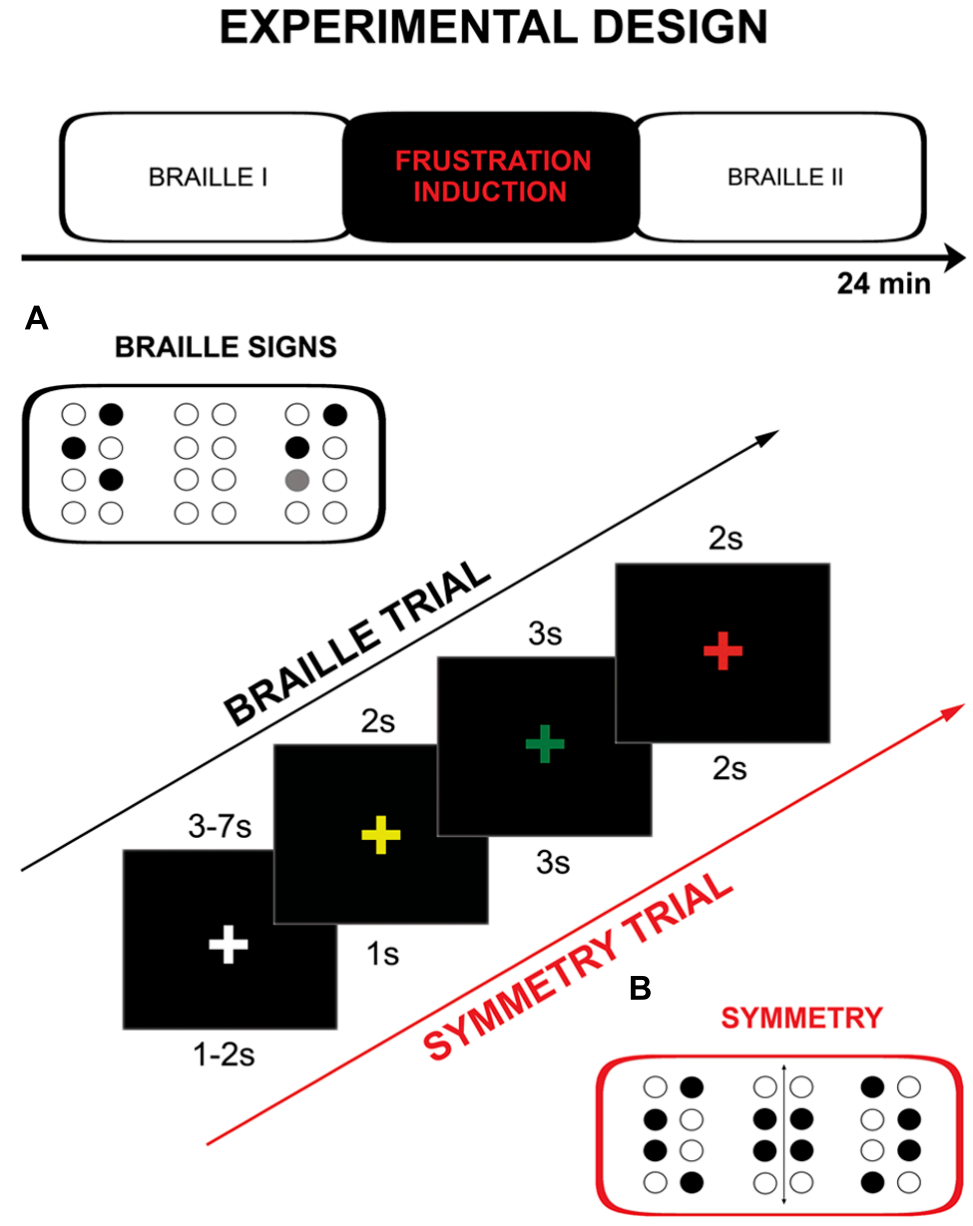
**Schema of the protocol including: experimental design, single trial timings and example of the used stimuli: **(A)** Braille characters, **(B)** symmetry pattern**.

The fMRI procedure consisted of three parts: two runs of tactile discrimination of Braille signs (Braille I and Braille II) separated by frustration induction (**Figure 1**). During Braille I and Braille II runs subjects were asked to compare two (the same-different) simultaneously presented Braille characters. One pair of signs was presented for 3 s. During the experimental session the subjects were asked to hold their right hand on the interface of the stimulator and touch its surface (pins array) only with the index finger. They were also requested to constantly look at the fixation cross (‘+’) located in the center of a black screen. The change of the fixation point color was used as a cue. The sequence of colors in a single experimental trial was following: yellow “+” is presented for 2 s as a ‘get ready’ cue, the “+” turns green and at the same time characters are displayed on the stimulator, after 3 s “+” turns red which is the signal to respond, “+” changes again to white for 3–7 s (jitter) rest interval. The constant display of a fixation cross that changed its color depending on the task was used as a way to keep the subject’s attention and reduce head movements. Subjects gave their answers by pushing an appropriate (same vs. different) button on a magnetic resonance (MR) compatible response grip with left hand (NNL Response Grip^2^; with the thumb finger when characters were the same, with the index finger when they were different). A total of 48 pairs of Braille characters were presented (half of which were the same) randomly in each of Braille sessions (I and II).

The frustration induction procedure was conducted between the two Braille parts. It was similar to the tactile discrimination task from the previous part, however, several significant modifications were introduced. Firstly, the subjects were asked to decide whether the presented signs were symmetrical or non-symmetrical instead of just assessing their identity. Secondly, the stimuli used were not typical Braille signs – they were not previously learned and meaningless. Thirdly, the trials in this part were presented at higher pace as cue presentation times were shortened: yellow “+” 1 s, “+” green for 3 s, “+” red 3 s, “+” changes again to white for 1–2 s (jitter). The last, and most important change, was introduction of the special instructions and negative feedback which were modeled after the MIST procedure (Dedovic et al., 2005). Instructions were presented before and after the training (“New task starts NOW. Your task will be to assess the symmetry of stimuli presented. Try to respond as quickly and as accurately as possible. If the number of correct answers is not sufficient enough, the test results cannot be calculated.”). Subjects were exposed to negative feedback after every six trials [“WARNING! Your level of performance is unsatisfactory. Try to do the task better!” (x3)]. At the end of the frustration induction procedure following information was presented: “Your level of performance IS EXTREMELY LOW AND DOES NOT ALLOW FOR THE CALCULATION OF THE RESULTS. Go back to the Braille discrimination task.” Frustration induction procedure consisted of 24 symmetry discrimination trials and training (two trials).

Experimental stimuli delivery, as well as behavioral response recording was controlled by Presentation^®^ (Neurobehavioral Systems, Albany, CA, USA).

### Pre-test and Behavioral Results

The frustration procedure was pre-tested in the pilot study (15 subjects) carried out outside the MR scanner. Two measurements were acquired during each session: (1) semantic differential affect rating scale (adapted from Bond and Lader, 1986) – repeated before and after the session, (2) galvanic skin response (GSR) recording during the session as an indicator of stress experiencing (Bakker et al., 2011). Six items were rated: nervousness, frustration, peacefulness, irritation, satisfaction, disorientation. Participants reported significantly higher level of frustration and irritation after the experiment.

**FIGURE 2:**
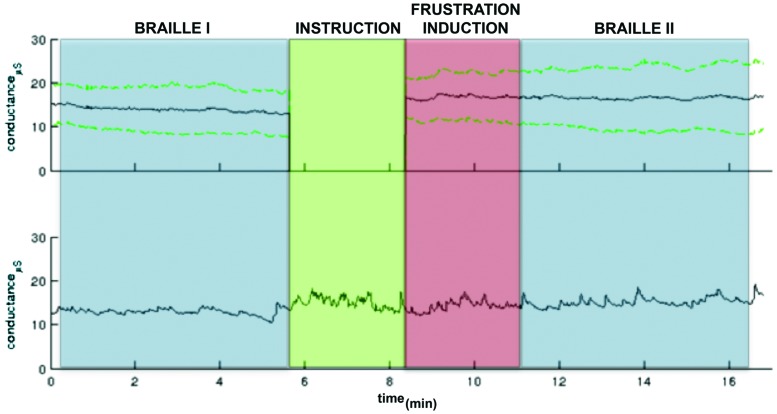
**A GSR observed in the pre-test pilot study.** The colors indicate particular part of the experiment: Braille I and II (blue), the instruction (green) following the frustration induction (red). The upper panel: the grand mean GSR (black line) and ± standard deviation lines (green dashed lines). The green part (instruction) was excluded from group analysis due to variable length. The activity only in particular task parts was calculated. The lower panel: An example GSR time course.

As far as GSR measurement, physiological monitoring system (BIOPAC Systems, Inc., Goleta, CA, USA) was used to record electrodermal activity sampled at 1 kHz. Preprocessing and GSR analyses were conducted in Ledalab^3^ After visual inspection three subjects were excluded from further analysis due to very low signal quality. Original signals were smoothed with a median filter in order to remove outliers and downsampled to 20 Hz. Subsequently Continuous Decomposition Analysis (CDA; Benedek and Kaernbach, 2010) was applied with GSR threshold amplitude set 0.05 μS. Resulting skin conductance response (SCR) and skin conductance level (SCL) separated for each block were statistically analyzed under MATLAB (Mathworks, v2011). Mean SCL were calculated for three parts of the procedure: Braille I, frustration induction and Braille II. Shapiro–Wilk test revealed non-normal data distribution in blocks, so statistically significant difference was found [χ^2^(2) = 7.2; *p* = 0.02] with the Friedman test. Wilcoxon test with Bonferroni correction for multiple comparison revealed that autonomic arousal during the frustration induction (*M* = 16.12, *SD* = 5.70) was significantly higher (*p* = 0.03) than in the Braille I (*M* = 12.36, *SD* = 5.01). No significant difference (*p* = 0.52) was observed between frustration induction and Braille II (*M* = 16.42, *SD* = 7.70) indicating that increased level of autonomic arousal persisted after the end of the frustration manipulation (**Figure 2**).

The level of task accuracy was also compared. One-way within subject ANOVA revealed significant differences between three parts of the experiment [*F*(2,56 = 55.8, *p* < 0.001, $\eta_{p}^{2}$ = 0.67]. Bonferroni *post hoc* tests indicated that in both Braille sessions average accuracy was significantly higher (Braille I: *M* = 81%, *SD* = 17%, Braille II: *M* = 84%, *SD* = 15%) than in the Frustration block (*M* = 51%, *SD* = 11%, both *p* < 0.001) with no significant difference between pre- and post-frustration performance.

### Image Acquisition and Data Analysis

Whole brain imaging was performed with a 3-Tesla MRI scanner (Siemens Magnetom Trio TIM, Erlangen, German) equipped with 32-channel phased array head coil. Head movements were minimized with cushions placed around the participants’ heads.

Functional data were acquired using a T2^∗^-weighted gradient echo planar imaging (EPI) sequence with the following parameters: time repetition = 3000 ms; time echo = 30 ms; flip angle = 90°; matrix size = 96 × 96; field of view = 190 mm; in-plane resolution: 1.98 mm × 1.98 mm; and 48 axial slices, with 3 mm slice thickness with no gap between slices. Detailed anatomical data of the brain were acquired with T1-weighted (T1w; time repetition = 2530 ms; time echo = 3.32 ms) sequences with isotropic voxel size (1 mm × 1 mm × 1 mm). For each subject, the functional run consisted of 480 volumes lasting 24 min. There were two main experimental conditions Braille I, Braille II with 48 number of trials and instruction between the blocks.

Statistical Parametric Mapping (SPM8, Wellcome Trust Center for Neuroimaging, London, UK) running on MATLAB R2012 (The Mathworks, Inc., Natick, MA, USA) was used for data processing and statistical analyses. First, functional images were motion corrected. Then, structural images from single subjects were coregistered to the mean functional image. High-dimensional Diffeomorphic Anatomical Registration through Exponentiated Lie Algebra (DARTEL) was used to create a group-specific template and flow fields based on segmented tissue from T1w images. The functional images were normalized using compositions of flow fields and group-specific template to a 2 mm isotropic voxel size. Finally, the normalized functional images were smoothed with a 5 mm isotropic Gaussian kernel. In the first-level statistical analysis for each subject experimental runs were split into single model. All the experimental events in block conditions (Braille I, Braille II, and Instruction) were designed as well as head movement parameters entered as covariates into the design matrix. Each stimulus was modeled, starting when stimulus was presented and ending when it disappeared from the screen. All stimulus functions were convolved with the canonical HRF basis function. In a second-level group random effects analysis *t*-tests were computed for whole group and between the groups respectively.

All the reported data were family-wise error corrected (FWE) for multiple comparisons at the cluster level, and a significance threshold of *p* < 0.05 was applied. Only the main peaks of activation with a *t*-value within each cluster and their corresponding brain structures were reported. The number of voxels activated in significant clusters and its coordinates are presented in the **Table 1.**

**Table 1:** Areas significantly more activated after frustration induction during Braille characters discrimination task (Braille II vs. Braille I) for the whole group and for the low and high tolerance for arousal groups.

				Whole group

			**Coordinates**				
					
		**BA**	***x***	***y***	***z***	**T (peak)**	**Cluster size (k)**
R	Putamen	13	20	10	-3	6,14	253
	Caudate		18	12	12	5,53	
	Putamen		18	10	-12	4,84	
	Insula		36	-2	9	4,68	
			34	4	3	4,02	
L	Postcentral gyrus/SI	7	-46	-44	54	4,81	249
			-42	-40	48	4,7	
L	Middle frontal gyrus	46	-42	44	15	4,93	130
			-38	58	6	4,35	
			-32	46	15	4,29	
R	SII	22	58	-2	6	5,93	121
			62	4	12	4,32	
R	Postcentral gyrus/SI	40	42	-46	48	5,24	110
R	Posterior cingulate cortex	23	8	-34	27	5,31	107
			2	-24	27	4,42	
R	Inferior frontal gyrus	44	50	10	21	4,97	75
			48	4	24	4,88	
R	Precuneus	7	14	-64	54	4,86	62
L	Putamen	13	-20	12	3	5,37	61
			-24	4	-3	3,73	
R	Middle frontal gyrus	10	40	46	6	4,36	58
			40	46	15	3,88	

**Low tolerance for arousal vs. high tolerance for arousal**							

L	PCC/precuneus	23	-2	-40	24	5,03	69
			-2	-48	39	3,92	
L	Inferior parietal lobule	40	-50	-60	45	4,64	70
			-56	-54	45	4,24	


*MNI coordinates, Brodmann areas (BAs), peak activation and volume are presented at the estimated significance threshold. t-values and cluster size (k), *p* < 0.001 (correction for multiple comparisons at cluster level). SI, primary somatosensory cortex; SII, secondary somatosensory cortex; PCC, posterior cingulate cortex.*

## Results

### Whole Group Analysis

**FIGURE 3:**
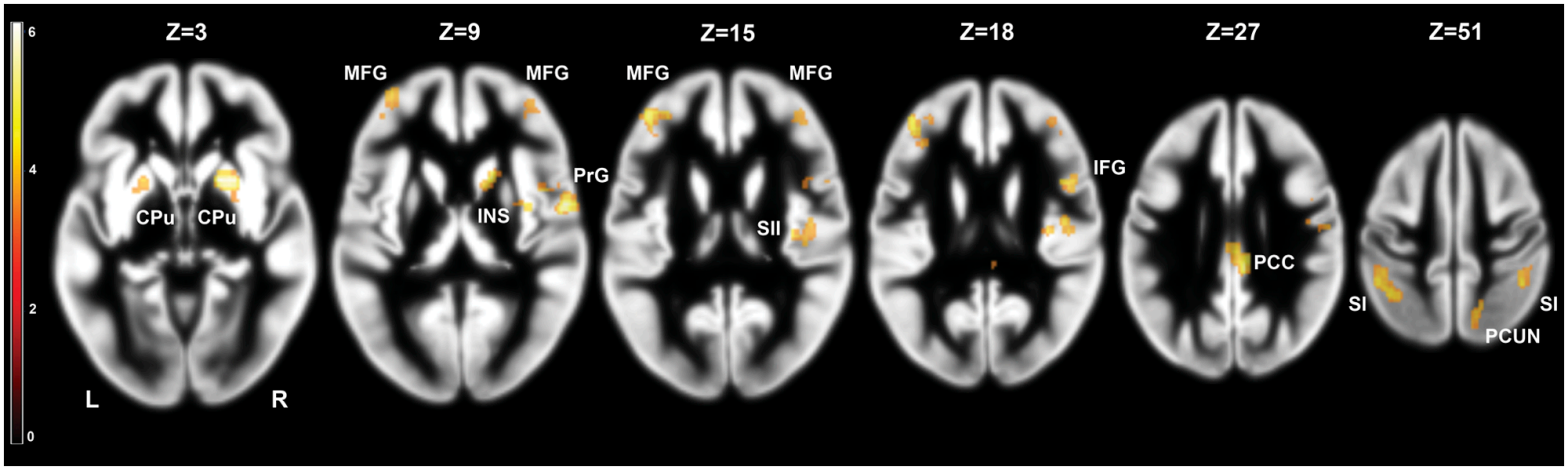
**Brain activity pattern in the whole group (*n* = 29) associated with the effect of frustration induction on Braille characters discrimination task (Braille II vs. Braille I).** CPu, caudate-putamen; MFG, middle frontal gyrus; PrG, precentral gyrus; INS, insula; SII, secondary somatosensory cortex; SI, primary somatosensory cortex; PCC, posterior cingulate cortex; PCUN, precuneus; L, left side; R, right side.

We investigated the effects of frustration on brain activation by contrasting pre- and post-frustration trials (Braille II vs. Braille I) in all the subjects using paired samples *t*-test. This comparison revealed significant differences in activation of basal ganglia, right insula, bilateral post-central gyrus, bilateral middle frontal gyrus, right SII, right posterior cingulate cortex (PCC), right inferior frontal gyrus, and right precuneus. Detailed results are presented in **Table 1** and **Figure 3.**

### Low vs. High Tolerance for Arousal

**FIGURE 4:**
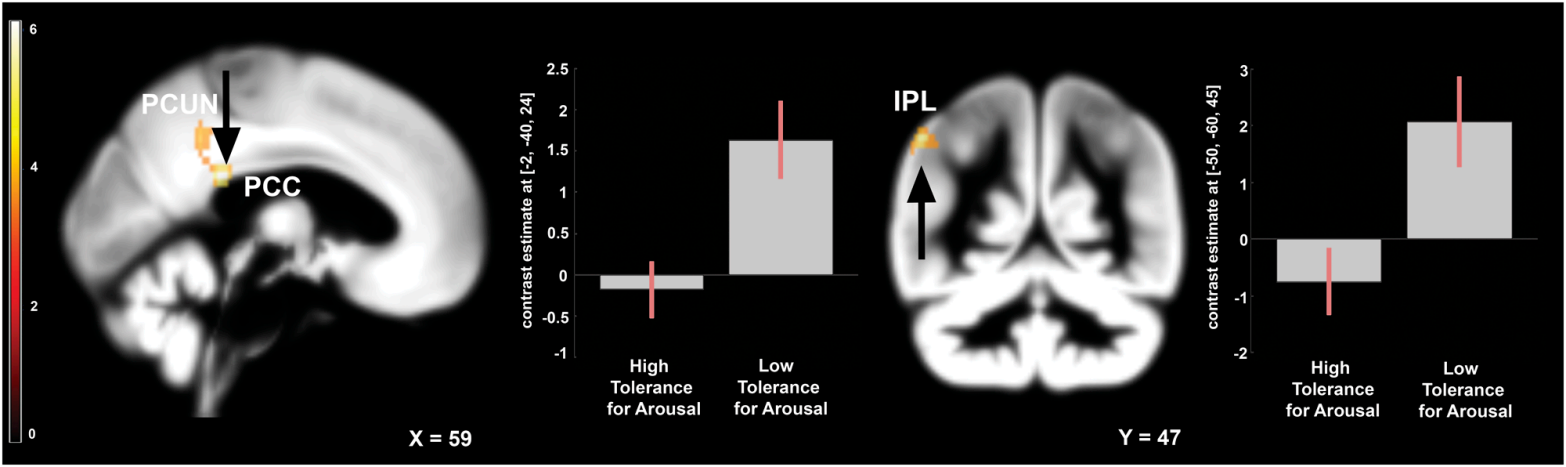
**Brain activity pattern between two groups: low arousal tolerance vs. high arousal tolerance associated with the effect of frustration induction on Braille discrimination task (Braille II vs. Braille I).** Plots represent percentage signal change for peaks in PCC and IPL. All of the displayed results are located in the left hemisphere. PCC, posterior cingulate cortex; PCUN, precuneus; IPL, inferior parietal lobule.

To address the issue of moderating role of temperament we compared the effects of frustration in groups with high and low tolerance for arousal. Independent samples *t*-test was applied to the contrast representing within-subject effects of frustration (Braille II vs. Braille I). This analysis revealed that in the low tolerance for arousal group frustration led to larger increase in activation of left inferior parietal lobule (IPL), left precuneus, and left PCC (**Figure 4**). Reverse contrast showed no differences. Detailed results are showed in the **Table 1.**

## Discussion

A new procedure of inducing frustration was introduced in the experiment. To the best of our knowledge this is the first study showing effect of frustration connected to performance of learned task – Braille reading. The participants learned a new skill – Braille characters recognition – specifically for the purpose of the study.

The frustration induction procedure was pre-tested in the pilot study outside the MRI scanner. We applied characteristics of stress inducing procedures (Dedovic et al., 2005, 2009) that meet the criteria of frustration. Braille reading was chosen because this activity has a well-examined brain activation pattern and this is not very common among sighted people, allowed us to control the level of performance. During 2 weeks of training and the experiment we tried to motivate subjects to perform the task as well as possible, as to make it their main objective. This action aimed to induce an atmosphere of high achievement. Negative feedback in frustration induction part was fixed and unrelated to actual level of performance. Negative feedback and new task made the impression on subjects of having no control over the situation which is an obligatory factor of frustration induction (Dickerson and Kemeny, 2004). The method we used in order to induce experimental stress refers to most basic definitions of frustration, which state that frustration appears with the omission of desired goal (Amsel, 1992). The pilot study confirmed that we managed to achieve this goal. Participants reported the feeling of frustration and annoyance after on the questionnaire during the pilot study. Furthermore, the GSR level during the frustration induction part increased significantly. Induced frustration did not reduce task accuracy in Braille discrimination task.

### Task Accuracy

In our paradigm performance depended both on successful retrieval of the knowledge acquired during Braille training and efficient processing of tactile information. The effects of stress on memory can be either positive (shortened reaction time Duncko et al., 2009) or negative (more mistakes, de Quervain et al., 2009). Some studies suggest that stress before learning of a word list enhances subsequent memory (Smeets et al., 2008; Schwabe et al., 2009), whereas other studies report that pre-learning stress impairs spatial or episodic memory (Kirschbaum et al., 1996; Elzinga and Roelofs, 2005). Hence, the impact of stress on memory abilities is diverse. Same is true for perceptual processes (Ileri-Gurel et al., 2013; Geva et al., 2014; Hoskin et al., 2014). The effects of acute stress seem to depend very much on exact task parameters measured and on experimental situation. In the case of our study, the behavioral results indicate that participants have mastered the Braille reading skills to the extent allowing them to cope effectively with the potentially disrupting effects of stress. At the same time, the difficulty of the task used to induce frustration lowered their accuracy to the guessing level (51% of correct answers), warranting credibility of the negative feedback information.

### fMRI Results

The fMRI experiment demonstrated that frustration can affect activation in structures which showed increased BOLD signal in acute stress studies: striatum, cingulate cortex, insula, middle frontal gyrus, and precuneus and in structures engaged in tactile discrimination task: SI and SII.

Caudate, putamen, and insula composed the biggest cluster of activation during the Braille discrimination task after frustration induction (compared to the same task before the frustration induction). It is known that dorsal striatum (caudate) activation contributes importantly to stress and negative affect processing (Dias-Ferreira et al., 2009; Seo et al., 2014). Furthermore, Schwabe and Wolf (2012) showed that stress alters the engagement of multiple memory systems in the human brain. They stated that stress impairs the hippocampus-dependent memory system and allows the striatum to control behavior. It results in a shift toward “procedural” learning after the stress appears. This kind of shift is proved to be adaptive, because it rescues the task performance after the stress (Schwabe and Wolf, 2012). This effect gives an insight into the lack of decrease in performance after the appearance of frustration observed in this study.

Another structure of the main activation cluster – the insula – is structurally and functionally connected to the basal ganglia, especially the caudate (Postuma and Dagher, 2006). In the experiment focused on neural correlates of frustration, insular activity, as well as that of inferior frontal gyrus, were interpreted as emotional pain processing (Abler et al., 2005). Both structures are also known to take part in negative affect processing (Wang et al., 2005), a state linked to frustration.

The use of fixed negative feedback applied in this study leads to another interpretation of basal ganglia activation – the dopamine reward prediction error hypothesis. Bush and Mosteller (1951a,b) proposed that reward prediction error is the difference between a weighted average of past reward and the reward that has just been experienced. When those are the same, there is no error, and the system does not learn (Glimcher, 2011). In the study all of the participants attended 2 weeks of Braille reading training. After these 2 weeks two Braille letters discrimination was quite simple task for them (81 and 84% accuracy in Braille parts). When new task was introduced (symmetry discrimination) and negative feedback applied, prediction error may have occurred. In the reinforcement learning theory (Holroyd and Coles, 2002), striatal activity has been linked to error and feedback-induced learning. Similar to Robinson et al. (2012) we suppose that fixed negative feedback produced prediction error signal within the striatum.

On the other hand, cingulate cortex, especially PCC is proved to be activated during stress rumination (Soares et al., 2013). Therefore, its activation during the post-frustration Braille discrimination task may be highly expected.

Performing the well-learnt discrimination task under stress resulted in higher activation of primary and secondary somatosensory cortex. These regions are involved in this discrimination also in no-stress conditions (Debowska et al., 2013). We interpret this heightened activation as a result of increased attentional effort, triggered by the frustrating experience of the previous part of the experiment. As the increased attention was shown to modulate the response to touch in somatosensory cortex (Mima et al., 1998), we interpret this change as an effective adaptation to stress allowing participants to retain high levels of performance.

### Frustration and Temperament

Besides the impact of frustration on well-learned task we were interested whether temperament modifies brain activation pattern in response to stressor. Therefore we compared groups defined on the bases of high vs. low tolerance for arousal. This contrast revealed higher activity in the IPL, PCC and the Precuneus among the low tolerance group (low results in endurance, activity and high and emotional reactivity score).

Posterior cingulate cortex and precuneus are the main components of the Default Mode Network (DMN; Raichle et al., 2001). During goal-oriented activity, the DMN is deactivated. Activation of this network corresponds to self-referential thought and introspection (Raichle et al., 2001). Activations of PCC, precuneus and inferior parietal regions after stressor are commonly associated with longer processing of emotionally salient stimuli (Maddock et al., 2003; Wagner et al., 2005; Soares et al., 2013). On the basis of DMN literature, PCC and PCu activity should be decreased while performing goal oriented behavior (Raichle et al., 2001). Our results revealed higher activation of these structures after the stressor in low tolerance group. This finding suggests that increased or decreased activation of the PCC in a stressful situation may be a functional neural correlate of an individual’s tendency to show exaggerated or attenuated emotional reactivity.

Furthermore, feedback that was used in our study had evaluative character. This kind of feedback is proven to induce activation in structures connected to self referential thoughts (Pan et al., 2009) and may strengthen the temperamental effect on the DMN structures. At the same time IPL activation may be due to somatosensory information processing, as it was shown to be activated during two-point discrimination (Sadato et al., 2002) and during maintenance of tactile information for subsequent object discrimination (Stoeckel et al., 2004).

Proposed interpretation of the results concerning the effects of frustration rests on the assumption, that observed changes in activation patterns cannot be attributed to the effects of practice, i.e., repetition of the Braille task. Design limitations of the study (lack of non-frustration control group) does not allow to empirically test this alternative. However, in the context of the obtained activation patterns this explanation does not seem to be plausible. With prolonged task exposure during the experimental session we would expect to observe decrease in the intensity of emotional response and lower involvement in the task performance. As observed results were exactly opposite, interpretation relating to frustration (and not mere practice effects) is much more convincing.

## Conclusion

We found that frustration and stress during performing a well-trained perceptual task do not impair the level of accuracy, but this happens at a cost of increased brain activation, encompassing the basal ganglia together with the insula and somatosensory cortices. Temperamental differences play a role in coping with this situation, as subjects with low tolerance for arousal showed increase activation of structures involved in processing the subjective effects of stress.

## Author Contributions

Substantial contributions to the: conception or design of the work: MB1, MB2, WD, AN, JS, MK; acquisition: MB1, AM, WZ; analysis: MB1, MB2, AM, AD; interpretation of data for the work: MB1, MB2, AM, WD, MF, AN, MK, Braille stimulator control code: MF.

Drafting the work: MB1 revising it critically for important intellectual content: MB2, AM, WD, AD, WZ, MF, AN, JS, MK.

Final approval of the version to be published: MB1, MB2, AM, WD, AD, WZ, MF, AN, JS, MK. Agreement to be accountable for all aspects of the work in ensuring that questions related to the accuracy or integrity of any part of the work are appropriately investigated and resolved: MB1, MB2, AM, WD, AD, WZ, MF, AN, JS, MK.

## Conflict of Interest Statement

The authors declare that the research was conducted in the absence of any commercial or financial relationships that could be construed as a potential conflict of interest.

## Footnotes

1 http://www.pzn.org.pl/.

2 http://www.nordicneurolab.com/products/ResponseGrip.html

3 http://www.ledalab.de.
